# The importance and effectiveness of nutritional counselling in patients with autoimmune thyroid diseases in Poland

**DOI:** 10.1186/s12962-022-00397-6

**Published:** 2022-12-05

**Authors:** Ewa Czubek, Klaudia Alcer, Mirjana Varjacic, Piotr Romaniuk

**Affiliations:** 1 Department of Health Policy, Medical University of Silesia, Katowice, Poland; 2 Doctoral School, Faculty of Health Sciences, Department of Health Policy, Medical University of Silesia, Katowice, Poland; 3 Department of Fertility Pathology, Faculty of Medical Sciences, University of Kragujevac, Kragujevac, Serbia

**Keywords:** Diet, Hashimoto disease, Graves disease, Thyroid disease, Education, Dietary behaviour

## Abstract

**Background:**

Autoimmune thyroid diseases are the most common diseases in humans. Their pathogenesis is complex, pushing patients to search different ways of alleviating their effects, one of which is diet change. The aim of this study was to assess the role of medical personnel in shaping eating habits in patients with autoimmune thyroid disease based on experiences declared by patients.

**Methods:**

We examined 208 individuals, of which 205 were qualified for final investigation. We selected study participants using accidental sampling, based on their appearance in health care facilities, including the ones providing endocrinological advices. The relationships between the qualitative features were tested using the Chi-square test of independence, assuming the significance level of p < 0.05. In the case of the expected cardinality  < 10, the Chi-square test with correction for Yates continuity was used, while for the cardinality  < 5—the Fisher exact test.

**Results:**

People with thyroid disease are statistically more likely to use dietitian advice than people without thyroid disease. The highest percentage of respondents decided to modify their nutrition due to their own initiative. In addition, patients with autoimmune thyroid disease are statistically more likely to consider changing their diet to improve their well-being. The connection between the source of advice and modification of eating behaviour was also noted.

**Conclusion:**

Thanks to the joint effort of medical staff, patients can receive reliable knowledge about their disease, treatment and nutrition adapted to their needs.

*Trial registration* approved by the Bioethics Committee of Medical University of Silesia in Katowice (opinion no.: PCN/0022/KB1/80/2)

## Background

Autoimmune thyroid diseases (AITD), including Hashimoto's thyroiditis (HT) and Graves’ disease (GD), are among the most common autoimmune diseases in humans. Both are primarily caused by abnormal T cells. The first produces anti-thyroglobulin (anti-TG) and anti-thyroid peroxidase (anti-TPO) antibodies, and the second produces pathognomonic anti-thyroid-stimulating antibodies (TRAb). There is also postpartum thyroiditis, which occurs when the disturbed levels of thyroid hormone (euthyroidism) occurring during pregnancy do not return to normal levels year after delivery and affects almost 10% of women [1]. Hashimoto’s thyroiditis and Graves’ disease are estimated to occur in 5% of the population, half of whom are unaware of it. Women are 5–10 times more likely to get HT and 6 times more likely to get GD than men. In Poland, as estimated, the issue affects even approximately 22% of the population. A large increase in the morbidity of these diseases may be the result of the occurrence of sufficiently rapid changes in the environment, such as: transition to more hygienic living conditions, increased intake of iodine in the diet or radiation. Other triggers for autoimmune thyroid disease include stress (especially for GD), lithium treatment, hepatitis C infection, and smoking (for GD only) [2], [3]. In regions with sufficient iodine supply, the frequency of occurrence anti-TPO or Tg autoantibodies is approximately 15–25%, increases with age, and is highest in women [4].

Currently, no specific dietary guidelines or recommendations for people with autoimmune thyroid disease exist. However, ensuring adequate amounts of omega-3 essential fatty acids for their anti-inflammatory properties, as well as dietary fiber to prevent constipation used to be advised to patients. Additionally, both the quantity and quality of food are important in these diseases. Restricting it reduces the activity of the hypothalamic–pituitary–thyroid axis, reducing the levels of circulating thyroid hormones. In the case of patients with hyperthyroidism, it is necessary to increase the caloric value of food, by average 20–25% in relation to the normal demand. The supply of protein in the diet should also be increased due to the dominant catabolic processes [5]. Certain nutrients for the optimal functioning of the thyroid gland are of importance, and they should be sufficiently supplied in the diet. These are in particular selenoproteins, due to their protective value for the thyroid against damage by reactive oxygen species and inflammation, and increase immunological tolerance [6], also iodine, which is an essential micronutrient in the diet required for the functioning of the thyroid gland and the synthesis of thyroid hormones. The consumption of this micronutrient both below and above the recommended level is associated with an increase in the number of circulating antibodies [7]. Another important nutrients are iron, which deficiency impairs thyroid metabolism and affects the synthesis of thyroid hormones [7], vitamin D, which deficiency, according to studies, is linked with thyroid autoimmunity [8], goitrogeny, which have a negative effect on the functions of the thyroid gland by disrupting iodine metabolism and inhibiting the synthesis of thyroid hormones [9] and also lactose and gluten in context of gastrointestinal disorders, such as celiac disease, atrophic gastritis, lactose intolerance and Helicobacter pylori infection, since many of them can inhibit the absorption of levothyroxine used to treat the thyroid gland.

The responsibility of given categories of medical staff with regard to nutritional education and counselling in Poland has been defined in the national law. In case of physicians these regulations are placed in the Act on the professions of doctor and dentist (Journal of Laws 1997 No. 28 item 152), stating that nutritional counselling is part of the services that the doctors may provide, both in form of recommendations appropriate to the given disease entity, as well as a referral to visit a dietitian.

Nurses also are responsible for health education and health promotion, including counselling related to nutritional behaviours. So is the case of midwifes, especially in case of pregnant women, including those suffering from thyroid disease, but also in the possible occurrence of postpartum thyroiditis (Journal of Laws 2011 No. 174 item 1039).

Dietitian, according to the European Federation of the Associations of Dietitians (EFAD), is a qualified specialist in nutrition, educated to at least Bachelor level, who instruct individuals and groups about health and diseases. The role and scope of responsibilities of this profession has not yet been clearly regulated in the national law in Poland.

The aim of this study is to assess whether medical professionals are being recognized as important source of nutritional knowledge in patients and whether they can effectively impact the nutritional behaviours, based on the example of people suffering autoimmune thyroid diseases.

In this context we aimed at verifying

1. what are the preferred sources of information patients use to gain nutritional knowledge
2. what is the willingness of patients to use professional nutritional counselling
3. whether there are any differences in the quality of counselling provided by different categories of medical professionals and other sources of information
4. what is the efficiency of nutritional counselling, as perceived by patients.

## Material and methods

The study covered 208 individuals, of which 103 were people diagnosed with thyroid diseases and 100 individuals without this type of disease were a control group. We selected study participants using accidental sampling, based on their appearance in health care facilities, including the ones providing endocrinological advices. To collect data we applied questionnaire consisting of 18 questions, divided into two parts. The first part were 5 questions on the socio-economic status of the respondent (gender, age, occupational status, education and place of residence), while the second focused on disease suffered, dietary behaviours and the use of dietary counselling delivered by different categories of medical staff, including physicians, nurses and dietitians. We asked study participants about their perception of the counselling obtained, satisfaction and results of the dietary advising they received, as well as other sources of information they use to obtain knowledge about nutrition recommendations in their disease. The survey was conducted between 10.02.2020 and 10.05.2020.

The relationships between the qualitative features were tested using the Chi-square test of independence, assuming the significance level of p < 0.05. In the case of the expected cardinality  < 10, the Chi-square test with correction for Yates continuity was used, while for the cardinality  < 5—the Fisher exact test. The results were prepared with the use of Microsoft Excel Office 365. The statistical analysis was performed with the use of Statistica version 13.3.

### Ethical standards disclosure

This study was conducted according to the guidelines laid down in the Declaration of Helsinki and all procedures involving research study participants were approved by the Ethics Committee of Medical University of Silesia in Katowice (opinion no.: PCN/0022/KB1/80/2). The bioethics commission concluded that the study does not constitute a medical experiment. The subjects/patients, by filling in the questionnaire, gave us informed consent to their study participation.

## Results

The majority of the respondents were women (91.19%), which may result from the higher occurrence of autoimmune thyroid diseases among the female sex. In the studied group 27% of respondents suffered from Hashimoto’s disease, and 9% had Graves’ disease. Other diseases or their combination (simultaneous Hashimoto’s and Graves’ disease, postpartum thyroiditis, hypothyroidism, non-toxic nodular goitre, thyroid nodules, thyroidectomy, hyperthyroidism) was noted in 18% of respondents.

The purpose for diet modifications, reasons for that, types of recommendation for diet modification given by medical specialist and also implementation, period of use, effects of received recommendations, are presented in Table 1. All listed results were estimated for two groups: participants with and without thyroid disease. In general patients suffering thyroid disease are more likely to change their behaviour that the control group. They also more often declare the long-term effect of the change, as well as positive results of the change in terms of their health status and well-being. However, in most cases the difference between investigated groups were not statistically significant, with the only exception for duration of the effect of diet change application, where we observed better effect in case of people suffering from thyroid disease. A difference close to statistical significance appeared in case of declaration regarding whether the specialist recommendation was even applied into practice.

**Table 1:** Diet-change behaviours in groups with and without thyroid disease

	People with thyroid disease (N = 103) (%)	People without thyroid disease (N = 100) (%)
Modifying diet to improve health (p = 0.169)		
Yes, I already done this	42.7	30
Yes, I take that into account	44.7	54
No	12.6	16
Modifying diet to improve well-being (p = 0.2)		
Yes, I already done this	41.7	31
Yes, I take that into account	46.6	59
No	11.7	10
Reasons for diet modification (p = 0.091)		
Own initiative	66.7	76.1
GP doctor suggestions	3.2	4.5
Specialist doctor’s suggestions	10.8	5.7
Dietitian suggestions	1	3.4
Family/friends suggestions	7.5	8
Others	10.8	2.3
Types of recommendation for diet modification given by medical specialist (p = 0.962)		
General recommendations	25.2	23
Specific recommendations	3.9	3
Recommendation to visit a dietitian	3.9	2
No recommendations	67	72
Putting the recommendations into practice (p = 0.0568)		
Following all the recommendations	18.0	16.5
Following selected recommendations	39.3	24.7
Not following any recommendations	42.7	58.8
Duration of application the recommendations (p = 0.014)		
Up to 1 month	18.4	13.1
Up to 1 year	21.8	10.7
Over 1 year	10.4	6.0
All the time till now	25.3	23.8
Not at all	24.1	46.4
Effects of the implemented recommendations (p = 0.484)		
Health improvement	10.6	6.1
Well-being improvement	20.0	17.1
Health and well-being improvement	25.9	24.4
No changes	43.5	52.4

The most popular information sources with regard to diet in both groups were Internet sources (54.71% and 41.11% respectively). Health care specialists were preferred by the first group in 23.58% of cases and in the second group in 36.67% of cases, however patients suffering thyroid diseases visibly more often declared consulting dietitians, while the control group seemed to stick to physicians’ counselling, with particularly strong difference observable in case of General Practitioners. Nonetheless, in general the differences between people suffering thyroid disease and the control group were not statistically significant (p > 0.05) (Table 2).

**Table 2:** Sources of information in groups with and without thyroid disease

	People with thyroid disease (N = 212) (%)	People without thyroid disease (N = 90) (%)
GP doctor	2.36	15.56
Specialist doctor	12.26	16.67
Dietitian	8.02	2.22
Nurse	0.47	2.22
Midwife	0.47	0
Internet pages	25.47	8.89
Blogs run by people with a given disease	16.98	18.89
Blogs run by dietitians	12.26	13.33
Scientific journals	2.36	5.56
Popular magazines	7.55	10
Family/Friends	5.66	5.56
Others	6.14	1.1

When it comes to dietitian counselling in both groups, the respondents most often declared not using this type of guidance. Those who did, were twice as many in the group of people with thyroid diseases—31% vs.14% (p = 0.00368).

Among respondents using dietary counselling, 39.29% declared applying all the recommendations, and 50% of them declared applying selected recommendations. In case of 10.71% of respondents, no change in behaviour was declared. In case of respondents who received recommendations from a specialist doctor, 17.65% declared following them in full, 58.82%—in part, and 25.53% did not modify their diet at all. The likelihood of following dietary counselling was in a statistically significant scale higher in case of dietitian advices (p = 0.00; Table 3).

**Table 3:** Percentage distribution of responses about diet modification by source of advice

Advice source	Following all recommendations (%)	Following selected recommendations (%)	Not following any recommendations (%)
Specialist doctor NO; dietitian NO (N = 94)	5.23	14.89	79.79
Specialist doctor NO; dietitian YES (N = 28)	39.29	50	10.71
Specialist doctor YES; dietitian NO (N = 34)	17.65	58.82	25.53
Specialist doctor YES; dietitian YES (N = 18)	44.44	44.44	11.11

When it comes to satisfaction of the counselling obtained, the highest percentage of full satisfaction appeared in case of dietitians. Nonetheless, if to sum up the answers of “rather satisfied” and “fully satisfied”, the highest percentage appeared in case of scientific journals. The highest general level of dissatisfaction we found in case of GPs, while midwifes received highest percentage of “fully dissatisfied” answers. Interestingly, in case of midwifes the answers were the most polarized, with equal number of patients declaring full dissatisfaction and those declaring full or high level of satisfaction. There were no statistically significant differences between answers in group with and without thyroid disease (p > 0.05; Table 4).

**Table 4:** Percentage distribution of responses about satisfaction from dietary recommendations

	Fully dissatisfied (%)	Rather dissatisfied (%)	Moderately satisfied (%)	Rather satisfied (%)	Fully satisfied (%)
GP doctor	43	16	22	16	3
Specialist doctor	20	22	20	23	15
Dietitian	20	11	20	13	37
Nurse	33	0	17	50	0
Midwife	50	0	0	17	33
Internet pages	11	19	24	35	11
Blogs run by people with a given disease	13	17	22	29	18
Blogs run by dietitians	13	16	28	23	20
Scientific journals	13	9	20	28	30
Popular magazines	21	24	27	21	6

## Discussion

As many other diseases, thyroid diseases contributes to the financial burden lying on the health sector. Costs of ambulatory and hospital care are significant and if to consider the decreased socio-economic activity, chronic diseases become even heavier burden for the national economies. [10]. The case of thyroid diseases is no different. Reports from Korea show that indirect cost of illness (COI) of thyroid diseases, including future income loss due to premature deaths and productivity loss resulting from work absence, in people in economically active age (in their 30 s, 40 s and 50 s) are accounted for nearly 25% of the total COI. Additionally, compared to other major diseases, the thyroid diseases showed a rapid growth rate in the first decade on XXI century in almost all cost categories, showing overall increase of 3.4 times between 2002 and 2010 [11]. In Ukraine total 12-month COI of hypothyroidism per patient was $868.26, and total 12-month COI of diffuse toxic goiter per patient was $1130.01, of which more than 65% were indirect costs [12]. The increase in costs is also notable in Denmark, where the total direct medicine and hospital care costs of thyroid diseases treatment per 100,000 persons increased between 1995 and 2015 by EUR ∼80,000 [13]. Estimations from the US regarding hypothyroidism-related total medical costs ranged from $460 to $2.555 per patient per year. In group of adult women in 2008 the spendings on treatment for thyroid diseases were of $4.3 billion. The reports emphasize also patients’ diagnosed with hypothyroidism impact on others patients, payers, and employers [14, 15].

Autoimmunology diseases, including thyroid diseases, are problematic when it comes to diagnosis and treatment. The disease initially evolves in a latent, but at this clinically asymptomatic time the destruction of individual organs proceeds and only as a consequence of the chronic process a full-blown form of the disease appears [16]. These issues might be less severe in case a common diet modification, due to both its primary preventive value, and ability to alleviate the effects of the disease. Overweight has negative impact on thyroid stimulating hormone (TSH) and thyroid echogenicity, and a correct diet-based therapy may limit this effect and normalize hormone levels [17]. All this makes the effective lifestyle dietary counselling potentially crucial factor determining the course of the diseases and reducing its burden [16], both in terms of individual wellbeing, and in the cost-related and macro-economic dimension. Contrary to this, in Poland the system of dietary counselling is poorly developed. Dietitian as a profession is not thoroughly regulated in Polish health policies and the number of dietitians employed in public health care facilities is decreasing [18]. Additionally, as the current data from Supreme Audit Office show, waiting time for metabolic diseases treatment for children and adolescents was even one year and the basic screening tests for obesity were executed only in 60% children, which is 30 percentage points less than in 2013 [19].

The results we obtained show that people struggling with thyroid diseases, especially with autoimmune thyroid diseases, need nutrition information about their disease. A high percentage of respondents who decide to modify their diets on their own initiative clearly confirms, that the recommendations they receive are not sufficient. There is a gap in existing studies on nutritional guidance and its effectiveness in patients suffering from these types of diseases, but similar results were obtained in studies on other groups of patients. Arana et al. [20] Investigated type 2 diabetes patients receiving dietary advice from a nurse in form of a general healthy eating leaflet. The participants of the study reported the need for an individual approach and conversation that would help them make appropriate diet changes, adapted to their living conditions. Another study assessed the nutritional status of patients with various cancer types, with as many as 69% of them reporting great interest in obtaining nutrition guidance in their disease [21]. A study by Maneze et al. [22] in turn investigated the information-seeking experiences of type 2 diabetes patients and its impact on self-management behaviour. The study found that the main cause of confusion and distrust in this group of patients, were the inconsistent, insufficient and incomprehensible recommendations they got from healthcare professionals. This corresponds in some extent with our results, where relatively low satisfaction with the guidance received from health care specialists, and relatively low willingness to follow their recommendation at the same time were observed.

There may be several reasons for such situations. The first is dissatisfaction with the communication between healthcare professionals and patients, which seems widespread. Therapeutic communication with the patient, which responds to the basic emotional needs, brings many benefits, e.g. greater sense of security, better treatment results and care process, more patients’ willingness to cooperate in the treatment process [23]. Physicians perceived as empathetic showed better treatment adherence and higher level of satisfaction among their patients. A key factor facilitating patient’s engagement in the process of treatment and care is adequate time devoted to communication, as the studies confirm [24].

On the other hand, however, medical specialists often have to deal with complex nutritional needs of the patient, especially in the case of comorbidities, while having a limited time that they can devote to individual patient. Pachocka et al. [25] reported that the vast majority of primary care physicians (84.2%) do not have enough time to provide dietary advice. Additionally, half of them do not consider their knowledge in this area sufficient. But what is even more important, 48% of physicians declared that they do not believe in the effectiveness of dietary counselling. In another study examining GPs’ views on the nutritional care provision to patients with chronic diseases, some respondents showed interest in taking measures to improve patients’ nutritional behaviour, but noticeable percentage expressed lack of feasibility conviction and profitability of such efforts. This position was against the expectations of patients, who generally considered nutritional care and guidance as important, although the study drew attention to their limited capacity to apply behaviour changes. Limited time spent on dietary counselling during the visit, and also lack of appropriate nutritional education among physicians might be factors that have some impact on this observation [26]. Deficiencies in the nutritional and preventive healthcare knowledge among medical personnel were observed in a Polish study by Bator [27].

Although studies suggest limited capacity to change nutritional behaviours among the patients, which was also observed in our study, they express a clear need to obtain effective education in this regard. Salmasi et al. [28] found that they expect receiving information in a concrete, factual form, especially with regard to how the disease could affect them, their quality of life and the activities they like, as well as what can they do to help themselves to limit the negative health outcomes. Salmasi’s examined subjects felt that the perfect educational program should enable them to consult health education experts, preferably in real time conversation.

Actual effectiveness of health education intervention in patients suffering different diseases was evaluated in a number of studies, including the one by Ghisi et al.[29], Ozturk et al. [30] and Alikari et al. [31]. Usually, the studies confirm successful improvement of patients' knowledge, physical activity, food consumption and self-efficacy and quality of life, although the better knowledge tends to translate into an implementation of recommendations in only a limited scale, which again confirms our observation. Again communication skills among the staff responsible for such programmes implementation seems to play a crucial role, as evidenced by Adam et al. [32], who observed promising results in terms of changes in diet and physical activity while evaluating the use of a patient-centred communication approach (healthy conversation skills) of dietitians in supporting women in achieving optimal weight gain during pregnancy and good health behaviour. Good results were also reported in systematic reviews addressing interdisciplinary nutritional care for patients with diabetes. Where nutritional education was associated with a reduced risk of the disease. The study showed the interventions provided by dietitians to be more effective than those provided by non-dietitians [33]. Another review by Mitchell et al. [34] found that dietary consultation for adults in primary care settings appeared to be effective in improving diet quality, diabetes tests results, weight loss, and reduction of weight gain during pregnancy. These observations are in line with the one presented in our study, where both guidance satisfaction and behaviour change were tended to be more effective in case of dietitians compared to other categories of medical staff.

Overall, our study confirms that patients show great interest and willingness to change their eating habits when being diagnosed. They do not remain passive and look for specific dietary information. However, when preferring easily available sources over the reliable ones, they may receive recommendations that are inadequate to their health status, making them ineffective or even harmful. There is a clear need for systematic patients’ education, which might help them to understand the disease, follow medical recommendations, and effectively change dietary behaviours in a longer time perspective. Our study confirms other findings in terms of the need to implement professional nutritional counselling delivered by qualified staff and supplementing regular medical care provided by medical and nursing staff with a separate category of dietitian services, since medical professionals often lack the necessary time or specialized nutrition knowledge to be able to provide effective guidance.

Our study may potentially have important implications for health system design, but it is of contributory nature only and has clear limitations, first of which is relatively small and non-representative study sample. Although other existing evidence suggest that our findings may be relevant also for patients suffering from other diseases, the general number of studies in the field is limited. More of them in needed to provide clear recommendations for national health policies with regard to staff training in terms of dietary counselling, as well as regulation of the profession of dietitian and access to dietitian services, especially in case of patients suffering certain types of disease.

## Conclusions

1. Patients tend to seek for dietary information primarily on the internet. Although medical professionals are being declared as the second preferred source, their role seems to be inferior, especially in the case of those who are trained to serve this role in the first place. The probability of seeking knowledge from medical professionals is rising, once the patient is being diagnosed with the disease.
2. Despite the above fact, patients perceive dietitians as the most satisfying and reliable source of nutritional knowledge and also, they are most likely to follow the recommendations obtained in case of receiving the counselling from the dietitian, who appears to be the most effective in shaping the dietary habits. A critical gap appears to exist in case of dietary counselling coming from other categories of medical staff, especially general practitioners.
3. Although more studies in needed to verify if and how the nutritional knowledge is being collected by other groups of patients, having regard to the economic and therapeutic efficiency of dietary counselling, based on our results it should be recommended to provide wide and easily accessible dietitian services available for patients, primarily in primary health care settings.

## Data Availability

The datasets used and analysed during the current study are available from the corresponding author on reasonable request.
